# Parent/caregiver attitudes, motivations and behaviours in relation to alcohol use among offspring aged 13–18 years: a qualitative study

**DOI:** 10.1186/s12889-022-12992-6

**Published:** 2022-04-05

**Authors:** Siobhan Mitchell, Rona Campbell, Georgie J. MacArthur

**Affiliations:** 1 Child Mental Health, University of Exeter Medical School, South Cloisters, St Luke’s Campus, University of Exeter, Exeter, EX1 1TE UK; 2 Department of Population Health Sciences, Bristol Medical School, University of Bristol, 39 Whatley Road, Bristol, BS8 2PS UK; 3 Public Health Research, Department of Population Health Sciences, University of Bristol, Canynge Hall, 39 Whatley Road, Bristol, BS8 2PS UK

**Keywords:** Qualitative research, Alcohol, Adolescent, Parent

## Abstract

**Background:**

Parental alcohol consumption and alcohol-related behaviour play a critical role in shaping adolescent alcohol use, but comparatively little is known about the perspectives of parents regarding adolescent alcohol use from qualitative studies in England. This study aimed to explore parental views and attitudes towards alcohol use during adolescence, among their offspring and among young people in general.

**Methods:**

Twenty-three parents (21 mothers, 2 fathers) of children aged 13–18 years were recruited via schools, workplaces and community settings, predominantly in the West of England (*n* = 19) between 2017 and 2018. Data were collected via in-depth one-to-one interviews and analysed thematically, using an inductive, constructionist approach.

**Results:**

Five major themes were identified in the data: (1) the parental alcohol environment, (2) balance and acceptance, (3) influences of the parental approach, (4) boundaries and parental monitoring, and (5) wider influences shaping young people’s behaviour. Overall, parents were aware of the risks and consequences of alcohol use and the wide range of influences shaping drinking behaviour, and expressed broad disapproval of alcohol use among young people. However, adolescent alcohol use was viewed as inevitable, and set within a context of a tolerant drinking culture. Many parents therefore chose a balanced and reluctantly accepting approach. This approach was determined by weighing disapproval of drinking against consistency with wider culture and parental behaviour, support for autonomy of the child, and avoidance of social sanctions. Parents’ responses were also determined by a desire to protect the parent–child relationship, maintain an open, communicative and trusting relationship, and ultimately limit risk and minimise harm. Various boundaries and strategies were employed to this end, including care around role modelling, gradual introductions to alcohol, boundaried provision, clear risk reduction messaging and parental monitoring.

**Conclusions:**

Parents employ a range of mechanisms to reduce alcohol-related risk and to balance harms of alcohol use among their offspring against adolescent behavioural norms. A downward shift in community consumption and changing socio-cultural norms could alter the accepting context in which parents are required to navigate adolescent alcohol use.

## Introduction

In the context of a broad array of factors that shape adolescent alcohol use [1–5], the family plays a critical role. Parental alcohol consumption and parental alcohol-related behaviour are positively associated with alcohol use in adolescent offspring. This effect has been shown to be mediated, in part, by a range of factors including parental monitoring, parental supply of alcohol, early initiation of alcohol use, association with deviant peers, and quality of the parent–child relationship [6–10]. In addition, the indirect effect of observation and modelling of parental behaviour via the transfer from parent to child of cognitive influences such as motives, norms, attitudes and expectancies, has also been shown to shape the beliefs and motives of parental offspring [11, 12].

Qualitative data further highlight the factors shaping parental attitudes and behaviours to adolescent alcohol use. Studies indicate how parents choose approaches and strategies thought likely to minimise risk and harm among their children, often in response to social norms [13], or a desire to ensure that their child fits in with others and avoids social sanctions [14, 15]. Moreover, studies report parental disapproval around underage drinking, yet introductions to alcohol are generally made in the home among family [13, 16, 17]. Furthermore, up to one third of young people report parents as their main source of alcohol [18], and young people perceive their parents to hold comparatively permissive stances towards adolescent alcohol use, in contrast to those held around tobacco or drug use [16].

Recent data highlight a downward shift in adolescent alcohol consumption, particularly among those drinking the most and to a greater extent among those in mid-adolescence compared to early adolescence [19]. Studies have suggested that determinants of such declines may be changes in parental attitudes, parental monitoring and/or changes to the parent–child relationship over time [20, 21]. Nevertheless, over one third of young people aged 15 have reported drinking at least once per month and excessive alcohol use is associated with increased risk of a range of harms, including injury, violence, regretted or unprotected sex, neurological impacts and increased risk of alcohol problems later in life [22–25].

Since the views and attitudes of parents both contribute to, and reflect, drinking culture, and parent attitudes and behaviours play a critical role in influencing adolescent alcohol use [8, 21], there remains a need for an in-depth understanding of parental perspectives and the drivers of parental behaviour. Our previous research has focused on the views of young people and young adults and suggested that a multi-faceted approach, involving parents and young people, is needed to change drinking culture and reduce alcohol-related harm [16, 26]. There is also comparatively little evidence from the United Kingdom (UK) context to support the development of UK-based interventions. In this study, we aimed to develop insights around the views and attitudes of parents in order to contribute to the evidence base and to inform the development of interventions to prevent excessive alcohol use and related harms among young people.

## Methods

### Sampling and recruitment

Participants were eligible to participate in the study if they had a child aged 13–18 years. The study sample included 23 participants (*n* = 21 mothers, *n* = 2 fathers; none were guardians or caregivers; seventeen participants reported their ethnicity as White British, three as White Irish, one as Asian British Indian, one as Black British African, and one as Mixed Race Other). Participants reported variable levels of alcohol use, from abstinence to regular alcohol use. Fifteen participants were recruited in 2018 via workplaces (pragmatically selected as local academic institutions) and community centres, snowball sampling, a regional public involvement network and Twitter. Participants resided mostly in the West of England (*n* = 19), but owing to the use of snowball sampling, also resided in the East of England (*n* = 1), South of England (*n* = 1), West Midlands (*n* = 1) and the Republic of Ireland (*n* = 1). Eight parent/guardians of young people aged 14–15 were recruited via secondary schools (n = 2 urban, n = 1 suburban) in the West of England in 2017 as part of a separate study. No participants dropped out of the study. These participants were parents of students randomly selected from Year 10 who gave consent to participate separately in the study. Parents received a letter via the school inviting their participation and that of their child separately; for parents that took part, their child did not give consent in relation to their parent’s participation. The topic guide was consistent for all participants. Sampling, recruitment and data collection took place in 2018.

### Data collection

In-depth one-to-one interviews (on average 55 min in duration) were conducted in private spaces in workplaces, in participants’ homes, or over the telephone, guided by a topic guide. The majority of one-to-one interviews were conducted by GJM (PhD, female, broad interest in the research topic) with additional interviews conducted by SM (PhD, female). Recruitment and data collection continued until saturation was reached. Both interviewers had previous experience in qualitative research. Before starting interviews, participants were aware of the aims of the study and reasons for doing the research. Interviews were facilitated by a flexible topic guide, which included questions around general attitudes towards alcohol use; perceptions of young people’s drinking, perceived influences on and risks of young people’s drinking; views on their own child’s alcohol use; the role of friends; parental decisions and influences; and the role of the family. Participants’ views on interventions for young people were also explored, but are not covered here. Interviews were audio-recorded and transcribed verbatim. Transcripts and findings were not returned to participants for comment or feedback. All participants received a £15 gift voucher for taking part.

### Data analysis

The aim of the analysis was to characterise and capture the attitudes, beliefs and influences underpinning and shaping parental viewpoints and approaches towards adolescent alcohol use. Since experiences and meanings relating to alcohol use can be socially produced and reproduced, rather than being solely inherent in individuals, analysis also sought to examine and theorise how parents construct meaning and develop ideologies and practices around alcohol-related parent–child behaviour through interactions with the social world and socio-cultural or wider environmental context. Our analysis therefore took a social constructionist perspective [27, 28].

Data were analysed by thematic analysis at the latent level in NVivo 11 (QSR International, Brisbane) using an inductive approach, as outlined by Braun and Clarke [27]. The researcher immersed themselves in the data, reading and re-reading transcripts and conducting open line-by-line, data-driven, coding to identify codes, and organize or segment the data (GJM). These codes were progressively linked, refined and grouped into potential themes, by analyzing patterns and relationships in the data, and seeking to understand and reflect the assumptions, beliefs, ideologies and wider socio-cultural and environmental contexts underlying the behaviours, perspectives and approaches described. The researcher moved between data and codes, generating thematic maps and writing analytic memos and notes to explore emergent thoughts, ideas and concepts and to combine codes to overarching themes. Once generated, themes were reviewed and refined to ensure that they reflected the perspectives, ideologies and meanings in the dataset.

### Ethical considerations

Written informed consent was obtained from all participants prior to participation and the study was carried out in accordance with the Declaration of Helskinki and research governance principles. Ethical approval for the study was granted by the University of Bristol Faculty of Health Sciences Ethics Committee (Ref 61921 and 131443(8201)/2306).

## Results

The major themes that were identified in the data were: the parental alcohol environment, balance and acceptance, influences of the parental approach, boundaries and parental monitoring, and wider influences shaping young people’s behaviour.

### The parental environment

Most participants aimed for moderate alcohol use or less, and described their close friends as having similar intake and attitudes. Participants described their own alcohol use in the context of their lifecourse, often being shaped by their previous experiences, observations of others, and experiences within their family. In particular, participants described a turning point when they became a parent, which entailed responsibilities and duties, and a desire to avoid role-modelling of alcohol consumption. Parents were aware of their role in setting expectations and norms, and the difficulties of setting boundaries for their child if they were not matched by their own behaviour.

Obviously when I was a student I used to drink and before I had kids. I have got kids to look after. I can’t possibly be drunk because I have got to look after them. (VP5, F)

I was mixing with people that were not using it [alcohol] well. I took a decision, and my husband did as well, not to drink because we were quite concerned that if we were to continue to have alcohol and use it in a way that we would normally, with young children growing up, we didn’t want them to have those kinds of influences (VP1, F)

### Balance and realistic acceptance

Despite the broad disapproval of adolescent alcohol consumption and awareness of its risks, many participants reported a desire to achieve a balanced and tolerant approach towards adolescent alcohol use. The foundations of such an approach included an acceptance of the inevitability of adolescent alcohol use, an understanding of the factors underpinning alcohol use, and a desire to avoid excessive alcohol use resulting from rebellion against a strict or abstinent approach. As such, a somewhat reluctant acceptance of the behaviour was positioned within a realism around the social, environmental and societal culture for adolescents. Forbidding alcohol use was viewed by many as likely to create a sense of excitement and appeal around alcohol use and thus a greater risk of covert and excessive drinking away from the home, which could lead to greater risk. Parents therefore aimed to foster more moderate or sensible approaches to alcohol use to minimise harm, while supporting their child’s social life and freedom to experiment. For some, such an approach followed a stricter method of managing alcohol use, which was subsequently seen as futile and damaging to the parent–child relationship and thus had been actively changed. For some, a balanced approach was necessary since parents themselves drank alcohol, which prevented them from forbidding alcohol use for their child.

I think if you’re not allowing them the freedom to do that, I think if you give them a little bit more freedom, they’re likely to stay within those boundaries, whereas if you give them a really tight boundary, they’re going to smash through it and go to the other extreme. (VP4, F)

Many parents sought to introduce alcohol gradually with clear limits to intake. Introductions were often made in the home on special occasions or at social gatherings where the child could sip a drink or try a small drink. Such introductions were driven by an interest in enabling the child to join in and to enable a controlled and supervised introduction to alcohol early in adolescence.

Well, I’ve always said to my daughter, “Do you want to taste this? Do you want to taste Prosecco?” New Year’s Eve she tasted Prosecco and was like, “Oh, no, I don’t like it at all.” Um, you can taste wine. So I think not making it taboo is really… is a really good way so they don’t think, “Oh, this is really something really exciting I’m not allowed to do.” Obviously, that’s going to make them want to do it. (R2, F)

Parents also reported mixing drinks for their child to take to parties or providing a fixed amount of beer or cider to be consumed, which was viewed as safer than refusal.

Nevertheless, some did report never providing their child with alcohol (see ‘boundaries’) and others reported not having alcohol in the home for young people to access or take to parties (overtly or covertly). Notably, a minority also described a firmer stance towards alcohol use by their child(ren), focused around abstinence.

I would say the last year, he said they were going to a party and he said, “Can I take some drink?” and I said, “No way, not at all.” He said, “My friend is,” and I said, “Well if your friend’s mum wants to give it to him, that’s up to him but there is no way I am buying alcohol for a 14-year old.” (VP11, F)

### Influences of the parental approach

Overall, the approach sought by participants appeared to be driven by aspirations of balance between broad disapproval of adolescent alcohol use, protection of the parent–child relationship, support and autonomy for the child, consistency with wider culture and parental behaviour, and a strong protective instinct, which aimed to limit risk and rebellion and thus minimise harm. Instinctive disapproval of adolescent alcohol use was tempered, in part, since an open, communicative and trusting relationship was viewed as critical to create a safer context around alcohol use and to maintain the quality of the parent–child relationship. Open, non-judgemental discussions facilitated awareness of the activities and whereabouts of their child, maintained an approachable stance in case of any problems and enabled parents to communicate safety messages.

They’re very, very open with me. As far as I know. Obviously, I think there are some things that they keep. They know I’m not going to freak out if they do tell me what has gone on. “Someone had this,” or, “They tried that.” So, I do think they tell me pretty much most of what they’re up to. (VP15, F)

The protective role of parents was evident via articulation of the importance of ensuring that they were physically and mentally ‘present’ with their children, taking time away from busy schedules to do things together, provide support, and listen.

Yes, and knowing that he could talk to me about stuff. It’s definitely, definitely that. I mean, “I haven't got time!,” and yes. “I’m really tired,” as I open a bottle of wine. Yes, that. So, you know, I make sure that we do have activities that we do together. I’m taking him up to the [event] this year so we can just do stuff (VP6, F)

Safety messages were communicated in an ongoing and routine manner, often opportunistically using trigger events or news stories, with parents judging when their child was ready. The major concerns expressed by parents included intoxication and loss of control; impaired judgement; vulnerability and a lack of safety; injury, accidents and fights; and health risks; and many expressed particular concern around their daughters and their increased vulnerability, including to sexual assault, being attacked and/or spiking. Thus, most messages were focused around negative consequences, reducing risks and strategies for staying safe (e.g. staying with friends, knowing limits), being responsible and social pressures.

Always be in control. Make sure that you can get yourself home safely. Stay in groups. Always look after your own drink, don’t leave it unattended. Yes, all of the normal things I would say to them.. (VP14, F)

### Boundaries and parental monitoring

In addition to the messaging, support and focus on the quality of the parent–child relationship, participants described a range of boundaries around alcohol consumption, as well as strategies for managing adolescent alcohol use, used to assure safety. Such boundaries involved age at introduction, offers of alcohol in the home and limits to provision.

They don’t drink in the house unless it’s, you know, like with a Sunday meal or something like that, I wouldn’t have them sat around drinking, I wouldn’t allow that. (VP10, F)

Boundaries were determined by a pre-existing sense of what was acceptable, the stage of adolescence, the personality of the child and often, some bartering, negotiation and compromise. An approach which was effective for one child did not necessarily apply to another, depending on their nature, desire to fit in with others, friendship group, and the extent of their interest in alcohol. Boundaries were supported by key parental monitoring strategies including communication with parents of young people holding parties to assess risk, providing lifts to and from parties, and having fixed collection times.

I am quite strong with it and I think that I have to be with him, because if I gave him the all clear he would be on a right jolly with it because he is quite easily led as well. (VP11, F)

It is supervised, insofar as I can do it, where I will do the drop-off and the pick-up and that’s at a set time. You know, it’s not go off to a party and come home whatever you feel like it. (VP9, F)

Importantly, however, participants described a clear contrast between their own approach and that of others, with many expressing disapproval and shock around the perceived leniency and low levels of monitoring observed among other parents, while acknowledging their respect for autonomy in individual approaches. For instance, participants described parental absence from parties, the provision of spirits to young people, and a lack of awareness around their child’s activities. For some, this was viewed as being borne out of an assumption that such an approach supported their child, while for others, this reflected the spectrum of norms around alcohol use among different families. Notably, alcohol also appeared to be a subject around which peers who shared similar values and attitudes might differ in their approach.

..even with someone that you consider to share some of your views with, even then there’s massive gap here with alcohol. That maybe comes back to the fact that within their family it’s so normalised that they just assumed it's normal for their 12-year-old child to have a beer at their own party. (VP3, F)

The distinction between participants’ norms and those in the wider social context of their child presented challenges around boundary-setting and approaches taken, since differences could undermine their own approach. Despite many parents describing an absence of felt pressure resulting from such differences, some reported feeling pressured by others’ approaches and discomfort with the result. Those that had actively built, and communicated with, a close network of parents, were emboldened and strengthened in boundary-setting, since this enabled awareness of others’ limits (e.g. around provision) and a shared agreement of consistent boundaries, while facilitating a greater awareness of the activities of the child.

I got a bigger group of mums together and said, “Right. [Name of child] is coming back saying, ‘So-and-so’s mum allows her to have…’ Can we just meet regularly to have a consensus and do some ground rules so we’re all saying the same thing? And we’re all saying the same thing about pick up times from parties and all that kind of stuff.” (VP4, F)

### Wider influences on young people’s behaviour

The major influences on young people’s behaviour that participants described were centred around environmental and cultural structures and norms, the influences of friends and peers, and the impacts of wider family members and networks.

### Cultural, environmental and commercial context

Alcohol use was described as strongly embedded in attitudes towards fun and socialising across the lifecourse, and was viewed as integral in social activities across the UK, while being widely available, affordable, widely promoted and broadly presented as a norm. Parents noted targeted advertising and marketing, and the promotion of sweeter alcoholic drinks, which had implications in shaping drinking culture among young people. Many also made the distinction between alcohol consumption in the UK and alcohol use in other European countries.

Changes over time, from parents’ youth, were seen to have resulted in a shift in alcohol use behaviour to a pursuit or intention of intoxication and excessive drinking. Notably, however, some parents highlighted that their children were more aware of alcohol and its dangers and self-regulated their behaviour more than anticipated.

### Peer influence and pressure

Peer influence and peer pressure was viewed as synonymous, and a major influence on young people’s behaviour, with parents accepting the likelihood that many young people would naturally aim to belong in a group, to be like their friends and avoid standing out. Nevertheless, the extent of influence was clearly dependent on a young person’s personality and often minimised by their own strength of character and firm views and attitudes, or the nature of their friendship group.

There are people there who get absolutely blotted, but she just thinks it’s quite pathetic. (VP13, M)

They have a huge influence because it’s all peer pressure and looking good and not standing out from the crowd. They have got the ability to influence it probably the most, peers. (VP3, F)

In part, peer influence was experienced via social networking sites (SNS), the extensive engagement with which was viewed as a source of concern for parents. Young people’s use of SNS presented a stark contrast with their own experiences of youth, which were free of the pressures of posts about alcohol and other peers having fun, or threats of being recorded or photographed. Positive portrayals of alcohol on YouTube or SNS was viewed as an influence, as well as a challenge for parents in managing the effects, although the potential for embarrassment or records of activity whilst intoxicated was also viewed as a potential restraint on behaviour. Despite such concerns, the use of social media and technology did not emerge as a major concern in relation to alcohol, in comparison to concerns around safety, vulnerability and boundaries around consumption.

Views on the comparative importance of peers or family in influencing young people were mixed with some describing peers as having an equal or greater influence, with others noting the critical influence and framework that parents and families provide, for instance in affecting how a child responded to peer pressure.

Everyone says the peer group is the most important thing, and I think they are really important unless… unless the parents are stronger than… than the peer group, and in terms of being around all the time (R3, F)

### The influence of others

As described above, other parents could present challenges to participants, as a result of discordant rules and boundaries. Views on the role of wider family members was mixed. Those who drank frequently or dependently could be negative role models and the focus of discussion about negative consequences of alcohol use; or alternatively, relatives could role model alcohol use in a more positive way. Broadly, such influences formed part of the wider context from which parents felt a protective duty, aiming to manage or protect their child from such effects.

With one I got to the point where I was like, “I don’t want my kids seeing how she drinks, because I don’t want them thinking that’s normal. This is an issue.” (VP7, F)

## Discussion

This study aimed to explore parental views and attitudes towards alcohol use during adolescence, among offspring and among young people in general. Our findings demonstrate that parents were broadly disapproving about alcohol use and reported concerns around safety and risk, but sought to achieve a balance between their disapproval and anxiety around alcohol use and a view that drinking was inevitable and normative during adolescence. This approach often led to adoption of a more accepting stance than was instinctive, founded upon trust, communication and support, which aimed to maintain quality of the parent–child relationship, while avoiding social sanctions and minimising risk and harm.

Our findings support those of related qualitative studies conducted outside of the UK, which report parental disapproval of alcohol consumption during adolescence, but a preference for boundaried alcohol use and the setting of limits around drinking, owing to perceived difficulty in controlling alcohol consumption at this stage [15, 29]. Our findings also support studies highlighting the importance to parents of their child ‘fitting in’ with others and being accepted in social groups, leading parents to seek to minimise harm social and alcohol related harm by allowing consumption, but avoiding provision or limiting the amount consumed [15]. Lastly, other studies similarly demonstrate the importance among parents of building trust in their child, the use of a conversational approach, and a focus on maximising the quality of the parent–child relationship [29, 30].

The focus among parents on minimising harm by adopting a more accepting stance than was deemed instinctive or ideal, was, in part, a response to the perception of adolescent alcohol use as normative, and an inherent recognition that alcohol use is deeply embedded in the social structures of society in the UK. We have previously reported how young people internalise and enact peer and cultural behavioural norms and accepting family contexts, generating and sustaining a social world where heavy alcohol use is normative [26]. Moreover, such embedded drinking cultures have been described in the literature, both historically, and through the 2000s, where a new ‘culture of intoxication’, ‘determined drunkenness’ [31] and ‘calculated hedonism’ [32] were described, involving developments in the alcohol industry and behavioral and attitudinal changes [31] leading to excessive consumption being reported as a norm [33]. Alcohol consumption at higher risk levels has been reported across a diverse range of drinking occasions and across age, sex and socio-economic groups in Great Britain [34] and more recently, the UK and Ireland were the only European countries were no statistically significant reduction in alcohol consumption was observed due COVID-19 pandemic [35].

While such drinking cultures were or are not unique only to the UK [36–38], taken together, our findings suggest that peer norms among parents, the perceived norms of young people, and cultural and societal factors perceived by parents are also a key influence on parental behaviours, rules and boundaries. The effects and pressure of perceived norms has been reported by others [13, 14], while evidence also indicates a perception among parents that views among the wider community are more supportive to underage drinking and supply of alcohol than are their own [39].

Many participants in our study reported a preference for the introduction of alcohol in the home and/or provision of alcohol, to avoid rebellion or excessive drinking outside of the home, and thus viewed parental alcohol provision as a form of harm reduction, as reported in related studies [40]. However, such views contrast with findings from the literature. For instance, parental supply of alcohol has been shown to be positively associated with alcohol consumption in mid-adolescence, and binge drinking and alcohol-related harm later in adolescence [18, 41], with the risk of alcohol-related harm being 2.5-fold higher among young people whose parents supplied alcohol, compared to those who had no supply of alcohol. The risk of harm increased further to fourfold higher if alcohol was supplied by parents and others [41]. In addition, the indirect effect of parental consumption has been reported to be mediated in part via early initiation [8], while evidence indicates greater risk of later substance use and risk behaviour associated with early initiation of alcohol use [42].

The means of influencing such practices may therefore warrant further investigation to explore how best to ensure effective harm reduction for young people over the short and longer-term. For instance, ways of strengthening awareness of the impacts of alcohol provision, could be incorporated into single and multi-component preventive approaches, as suggested by others [17], while addressing wider socio-cultural influences on behaviour. Since beliefs about alcohol are formed well before adolescence [43], multi-component approaches that address societal norms could also play a role in reducing intergenerational transference of cognitions, motivations and expectancies [12], via a shift in attitudes and reduced parental drinking at a population level. Indeed, many parents noted their role in setting expectations and role modelling behaviour and anecdotally noted higher levels of consumption among young people whose parents drank to excess.

Interestingly, our findings suggest an interplay between trusting, communicative parent–child relationships, family closeness (e.g. parental attention and support, participation in joint activities) and parental monitoring. The latter involved knowledge of the whereabouts of children, assessments of the settings and perceived risks of individual parties, the active development of parental networks and the provision of lifts to and from parties. Evidence demonstrates that the association between parental and adolescent drinking is mediated, in part, by perceived parental monitoring and discipline [9], and while there is little strong evidence for an effect of family closeness, joint activities or support, one study has suggested that monitoring and family closeness may be related [44] and this may warrant further investigation.

Interestingly, we did not find clear evidence of a perception among parents that there was a downward shift in alcohol use, with many noting concerns around the scale of alcohol use and cultural acceptability. However, the harm reduction and safety messages communicated repeatedly by parents, alongside implementation of a range of parental monitoring techniques and disapproving attitudes, reflect evidence of more restrictive alcohol-related parenting and less tolerant views in the study sample, which supports published evidence. For instance, studies have suggested a shift to more restrictive alcohol-related parenting behaviour and lower tolerance to adolescent drinking; and changes in parental permissiveness and practice has been suggested to be one possible explanation of the decline in adolescent drinking [45–47]. In this way, our findings lend support to reduced tolerance and increased boundary setting as implicated in the downward trends observed.

Recent studies also highlight that the setting of parental rules concerning alcohol and parental disapproval are associated with lower odds of risky drinking [48], while indicators of parental monitoring, knowledge of offspring whereabouts and restrictive attitudes towards offspring drinking were associated with abstinence among Swedish adolescents [21]. Lastly, clear parental rules and parental control were included among social mechanisms postulated to affect adolescents’ low alcohol consumption in Sweden [20]. We cannot account for attitudinal and/or behavioural changes that may have occurred since 2018, but taken together, our study provides qualitative evidence supporting the use of various approaches to parental monitoring, boundaried provision and parent–child communication to manage alcohol-related risk and potentially contribute to reduced adolescent consumption.

### Strengths and limitations

Our study has several strengths, including the sample of parents having been recruited from a range of communities, who reported variable levels of parental alcohol use and family histories. The use of in-depth semi-structured one-to-one interviews also enabled the collection of rich, detailed data regarding individual perspectives and personal experiences, unaffected by social desirability of responses or norm perception in the group. In addition, parents discussed experiences with both sons and daughters from across the course of adolescence. Nevertheless, we note that were able to recruit only two fathers, and the number of participants from low socio-economic groups and Black, Asian and minority ethnic groups was limited. As such, our findings may not be applicable to all areas and groups. In addition, our data were collected in 2018 prior to the COVID-19 pandemic, and there may therefore have been substantial changes in attitudes, behaviours and parenting practices, as well as changes in the determinants of alcohol use, which have not been considered.

### Conclusion and ecommendations

Our findings suggest that parents navigate a complex interplay of individual, family and environmental factors to protect offspring from risk and harm alongside support for social and peer involvement, parent–child communication and perceived normative alcohol use during adolescence, leading to a balanced approach. Greater awareness of the adverse consequences of early initiation to alcohol and of parental supply, alongside correction of misperceptions around attitudinal and behavioural norms could support parents and caregivers to reduce alcohol-related risk among adolescents. Population-based approaches that lead to a downward shift in general consumption are also needed simultaneously to address role-modelling and the transfer of motivations and behaviours from parent to offspring.

## Data Availability

The dataset supporting the conclusions of this article is available in the University of Bristol data repository, data.bris, at https://doi.org/10.5523/bris.3nj4vkhwx1c0u267vnknub8a4y One dataset including eight parents/ guardians generated and analysed during the current study are not publicly available due to a lack of consent for this from participants.
